# Effect of diode laser on micro-tensile bond strength of hybrid ceramic restorations to dentin using one-step and two-step self-etch adhesives: in vitro study

**DOI:** 10.1186/s12903-026-09504-y

**Published:** 2026-08-10

**Authors:** Cherif H. Mansour, Tamer A. Hamza, Hussein R. Mohammed

**Affiliations:** 1 Faculty of Oral and Dental Medicine, Misr International University, P.O. Box 1 Heliopolis, KM 28 Cairo-Ismailia Road Ahmed Orabi District, Cairo, Egypt; 2 Faculty of Dentistry, Faculty of Dental Medicine, Badr University in Cairo, Boys, Al-Azhar University, Cairo, Egypt; 3 Faculty of Dental Medicine, Boys, Al-Azhar University, Cairo, Egypt

**Keywords:** Ceramics, Micro-tensile bond strength, Laser Irradiation, Self-etch adhesive, Dentin bonding

## Abstract

**Background:**

This study aimed to evaluate the effect of diode laser irradiation on the micro-tensile bond strength (µTBS) of hybrid ceramic restorations bonded to dentin using one-step and two-step self-etch adhesives.

**Methods:**

An in vitro study was conducted using 20 freshly extracted human permanent molars. Standardized Class I inlay cavity preparations were performed, and hybrid ceramic inlays were fabricated using Shofu Block HC (Shofu Inc.). Specimens were randomly divided into four experimental groups (*n* = 5 per group) according to the adhesive strategy and laser treatment protocol. Two adhesive systems were evaluated: Clearfil S3 Bond Universal (Kurary Noritake Dental Inc.), applied in the self-etch mode (SE1), and Clearfil SE Bond 2 (Kurary Noritake Dental Inc.), a conventional two-step self-etch adhesive (SE2). Each adhesive group was further subdivided intolaser-irradiated (SE1-L, SE2-L) and non-irradiated control (SE1-NL, SE2-NL) subgroups. Laser irradiation was applied using a 940 nm diode laser after adhesive placement and before light polymerization in the SE1-L and SE2-L groups. The inlays were bonded using a light-cure flowable composite resin under static load. Following storage and accelerated aging by immersion in 10% sodium hypochlorite for one hour, specimens were sectioned into 0.9 mm microtensile beams. Microtensile bond strength (µTBS) was measured using a universal testing machine. Failure modes were analyzed using stereomicroscopy, and representative fractured specimens from each griuo were qualitatively examined by scanning electron microscopy (SEM). Data were analyzed using the Mann-Whitney U and Fisher’s exact tests (α = 0.05). Randomization and assessor blinding were implemented.

**Results:**

Laser irradiation significantly increased the µTBS of both one-step (SE-1) and two-step (SE-2) self-etch adhesives. For SE-1, laser-treated specimens showed a median µTBS of 13.7 MPa (mean ± SD: 16.3 ± 5.6) compared to 7.7 MPa (8.8 ± 5.6) without laser (*P*=.047). Similarly, SE-2 groups exhibited median µTBS values of 22 MPa (21.3 ± 3.1) with laser and 12 MPa (12.5 ± 5.4) without laser (*P*=.028). However, no statistically significant difference in µTBS was observed between the two adhesive types, regardless of laser application (*P*>.05). Failure mode analysis revealed no significant differences among groups (*P*=.167).

**Conclusions:**

Diode laser irradiation enhanced the µTBS of both one-step and two-step self-etch adhesive strategies when bonding hybrid ceramic restorations to dentin. No statistically significant difference was observed between the two adhesive strategies following laser irradiation. Further studies are needed to evaluate the long-term durability and clinical relevance of this approach.

## Clinical implications

Diode laser irradiation applied before adhesive polymerization may enhance the bonding performance of self-etch adhesive systems when bonding hybrid ceramic restorations to dentin. This adjunctive protocol significantly increased the microtensile bond strength of both simplified one-step and conventional two-step adhesive strategies under the experimental conditions of this in vitro study. Although the present findings are encouraging, additional laboratory and clinical studies are required to validate the long-term effectiveness and clinical applicability of this protocol.

## Introduction

With the advancement of adhesive dentistry, there has been a growing demand for durable and esthetic indirect restorations, particularly hybrid ceramic restorations that combine favorable mechanical and physical properties [1]. Materials such as hybrid ceramics, designed for computer-aided design and computer-aided manufacturing (CAD-CAM), offer improved strength and resilience compared to traditional ceramics [2, 3], making them suitable for conservative restorations like inlays and onlays [4–6]. When paired with modern self-etch adhesive systems and resin cements, these materials enable effective tooth rehabilitation without relying on extensive core buildups or intraradicular posts [4]. This approach supports minimally invasive treatment of teeth with varying degrees of coronal damage, optimizing both function and preservation of tooth structure [7].

A critical challenge in bonding hybrid ceramic restorations to dentin lies in achieving a durable and reliable adhesive interface [8]. Simplified one-step self-etch adhesive systems, favored for their ease of use and reduced technique sensitivity, often incorporate hydrophilic monomers that increase water permeability, making the adhesive layer more prone to hydrolytic degradation in the oral environment [9]. Additionally, the polymerization of light-cured resin cements used to lute hybrid ceramic restorations can be compromised by the thickness and opacity of the material, limiting light transmission and reducing monomer conversion [10, 11]. Adequate light transmission through ceramic restorations has been shown to improve polymerization efficacy compared to dual-cure systems, underscoring the importance of restoration design and material selection [12]. Furthermore, the use of highly filled flowable composite resins in combination with light-cure adhesives may enhance the formation of an acid-base resistant zone, potentially reducing the risk of secondary caries and improving the longevity of the bonded interface [13].

Two-step self-etch adhesives are considered the gold standard for dentin bonding due to their optimized formulation and separate priming and bonding steps [14, 15], which promote better resin infiltration, stronger hybrid layers, and improved durability—critical for hybrid ceramic restorations like inlays and onlays [16]. Two-step self-etch adhesives have demonstrated superior and more consistent performance compared to simplified one-step systems; however, their technique sensitivity may limit clinical convenience [17, 18]. To overcome this, laser conditioning of dentin has been investigated; while results vary, diode laser irradiation of uncured adhesives shows promise by enhancing resin penetration and bond strength without additional primers [19]. This suggests that combining laser pretreatment with one-step adhesives could offer a simpler yet effective alternative to two-step systems. However, the success of this approach depends on adhesive type, laser settings, and dentin characteristics, underscoring the need for optimized protocols tailored to hybrid ceramic bonding [20–23].

Despite significant progress in adhesive dentistry and the development of advanced materials for indirect restorations, there remains a gap in the literature concerning how diode laser irradiation interacts with different adhesive strategies—specifically, one-step versus two-step self-etch adhesives—when bonding hybrid ceramic restorations to dentin.

The effectiveness of diode laser irradiation applied over uncured adhesive systems is influenced by several interacting factors, including laser wavelength and energy parameters, adhesive composition, solvent type and concentration, and the kinetics of solvent evaporation prior to polymerization [24, 25]. These physicochemical interactions may differ between simplified one-step and conventional two-step self-etch adhesive strategies because of the differences in solvent content, hydrophilicity, and adhesive permeability [26]. The present study was performed using extracted teeth without simulated pulpal pressure, an experimental condition that may influence solvent evaporation dynamics and adhesive behavior compared with vital clinical conditions [27]. Therefore, further investigation of these interacting variables remains necessary to optimize laser-assisted adhesive protocols.

This study aimed to evaluate the effect of diode laser on the microtensile bond strength (µTBS) of hybrid ceramic restorations bonded to dentin using one-step and two-step self-etch adhesives. The null hypotheses tested were that (1) diode laser irradiation of the dentin bonding agent prior to polymerization would not influence the microtensile bond strength compared to non-irradiated bonding; and (2) there would be no difference in microtensile bond strength between the two-step and one-step self-etch adhesive strategies.

## Materials and methods

This in vitro interventional study evaluated the effect of diode laser irradiation on the µTBS of hybrid ceramic restorations bonded to dentin using one-step and two-step self-etch adhesives. The primary outcome measured was µTBS, expressed in megapascal (MPa), obtained from microtensile specimens tested for each tooth.

Sample size was calculated based on effect sizes reported by Bottino et al. [2] and Maenosono et al. [9] with values of f = 2.36 and 8.59 for the two factors, respectively. Using an alpha level of 5% and a beta level of 20% (power = 80%), five samples per group were determined to be sufficient, totaling 20 samples. The calculation was performed using a statistical software program (IBM SPSS SamplePower Release 3.0.1; IBM Corp). The number of teeth per group is also in accordance with the Academy of Dental Materials guidance for adhesive-dentin µTBS testing where tooth is used as the statistical unit [28].

**Fig. 1:**
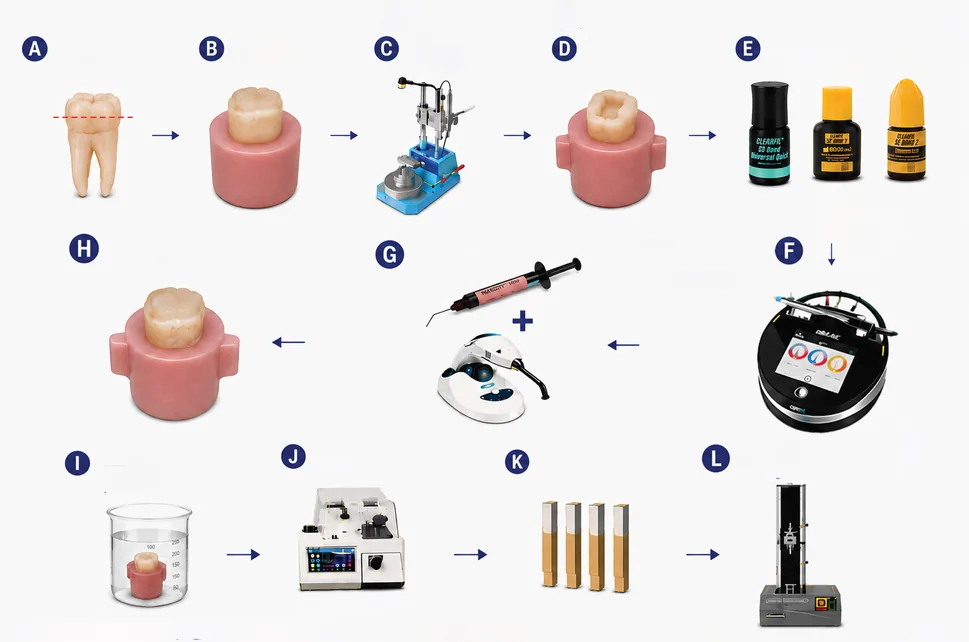
Schematic representation of the experimental workflow. Extracted human molars were randomly allocated to four experimental groups (*n* = 5/group) according to the adhesive strategy and diode laser irradiation protocol. Following specimen preparation, adhesive application, laser irradiation (where applicable), cementation, accelerated aging, sectioning into microtensile beams, and microtensile bond strength testing were performed as illustrated. (**A**) sectioning of occlusal one third to expose dentin; **(B**) Teeth with exposed dentin; (**C**) dental parallelometer; (**D**) Class I inlay preparation in teeth embedded in autopolymerizing resin; (**E**) application of adhesive strategy SE1 or SE2 according to grouping; (**F**) diode laser application to laser-irradiated subgrougs; (**G**) cementation; (**H**) cemented inlay specimen ready for sectioning; (**I**) aging by immersion in 10% sodium hypochlorite for one hour; (**J**) sectioning into microspecimens; (**K**) microspecimens; (**L**) microtensile bond strength testing

Twenty freshly extracted human permanent molars with comparable anatomical configuration were collected after approval by the Research Ethics Committee of Al-Azhar University, Faculty of Dental Medicine, Boys (ethically accepted with code 723/192). All procedures were conducted in accordance with the Declaration of Helsinki and the institutional ethical guidelines. Teeth were free of cracks, fractures, restorations, endodontic treatment, root resorption, and dilacerated roots. Remaining soft tissues were removed using an ultrasonic scaler. Teeth were disinfected and stored in distilled water at 4 °C for no longer than three months before use. A schematic representation of the experimental workflow is presented in Fig. 1A-D.

**Fig. 2:**
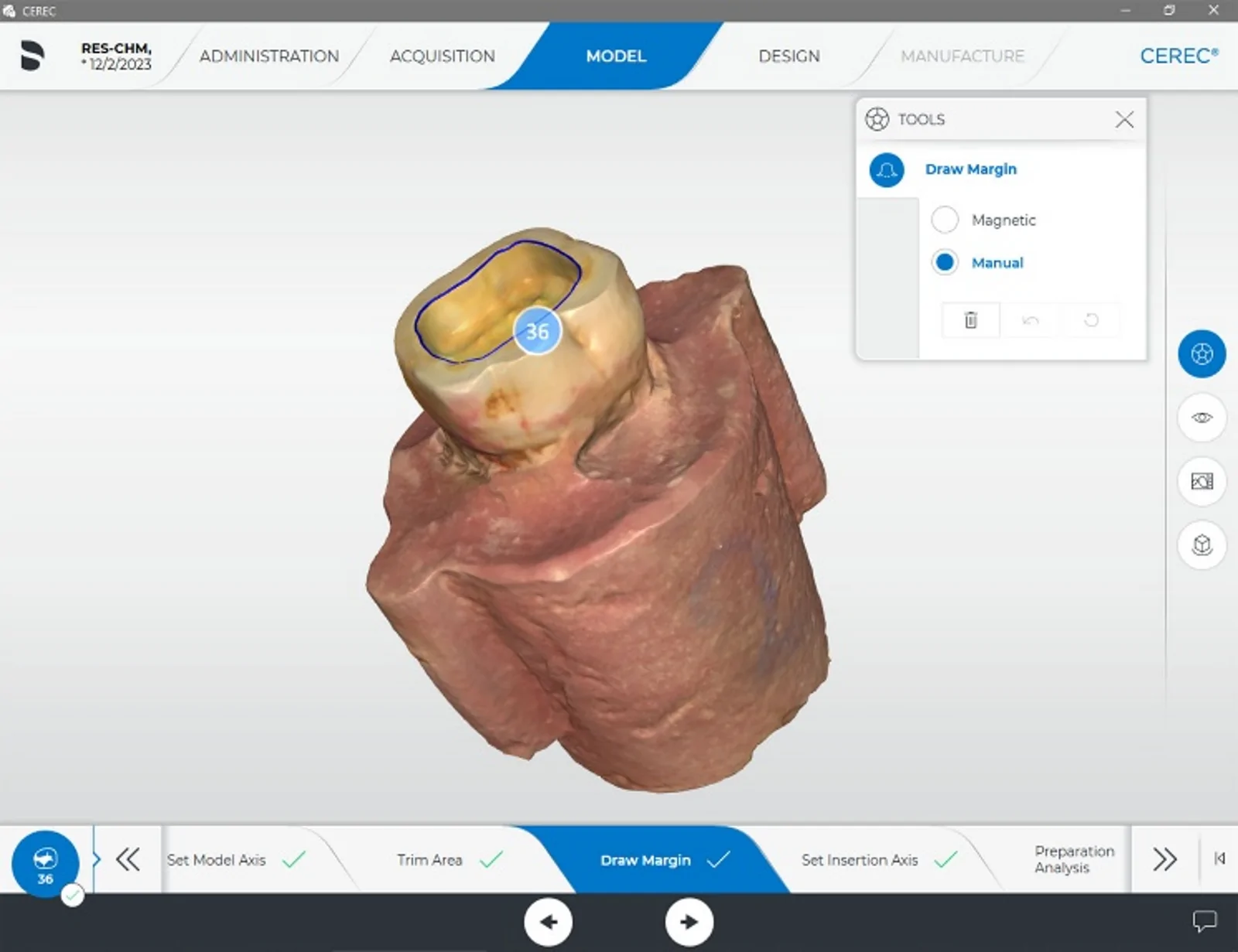
Scanned inlay cavity preparation

Each tooth was embedded in auto-polymerizing resin up to 2 mm below the cemento-enamel junction. The occlusal surfaces were flattened perpendicular to the long axis on the occlusal third to expose dentin by means of a sectioning machine (Isomet 4000; Buehler Ltd.). Standardized box-shaped Class I inlay preparations were made with round and conical diamond burs mounted on a dental parallelometer (AF 30 milling machine; Nouvag). Each cavity was prepared to dimensions of 8 mm mesio-distally, 6 mm bucco-lingually, and 2 mm in depth, featuring a 6-degree wall divergence and rounded internal line angles. The prepared cavities were subsequently scanned using an intraoral scanner (CEREC Omnicam; Sirona Dental Systems) (Fig. 2).

Hybrid ceramic inlays were fabricated from resin-matrix ceramic blocks (Shofu Block HC; Shofu Inc.) using CAD-CAM technology. The inlay restorations were designed virtually using a CAD software (CEREC SW 5.2; Sirona Dental Systems), where a cement space of 80 μm was programmed to ensure optimal fit and retention. Milling was performed using a 5-axis milling unit (MCX5; Sirona Dental Systems).

Samples were randomly allocated into two main groups according to the type of adhesive strategy used (*n* = 10 each); Group SE1: One-step self-etch adhesive strategy (Clearfil S3 Bond Universal; Kurary Noritake Dental Inc) and Group SE2: Two-step self-etch adhesive strategy (Clearfil SE Bond 2; Kurary Noritake Dental Inc.). Each main group was further divided into two subgroups based on the application of diode laser irradiation (*n* = 5 each); SE1-L: One-step adhesive strategy with laser irradiation, SE1-NL: One-step adhesive strategy without laser irradiation, SE2-L: Two-step adhesive strategy with laser irradiation, and SE2-NL: Two-step adhesive strategy without laser irradiation.

After cavity preparation, the dentin surfaces were gently rinsed and kept moist to prevent desiccation. Adhesive systems used, their compositions, and the application procedures performed in this study are shown in Table 1. For the SE1 group, the adhesive was actively applied onto the dentin using a micro-brush with continuous scrubbing for 20 s to ensure thorough infiltration, followed by gentle air-drying for 5 s to evaporate solvents and form a uniform film. For the SE2 group, the primer was first applied with agitation for 20 s, air-dried for 5 s, then the bonding agent was applied and similarly air thinned. In the laser-irradiated subgroups, a diode laser of 940 nm ± 10 nm (EpicX; Biolase) was applied to the adhesive-coated dentin surface before light curing to enhance adhesive penetration and interaction with dentin (Fig. 1F). Both adhesive systems were applied in accordance with the manufacturers’ instructions for use and established adhesive application protocols [29, 30].

**Table 1:** Adhesive systems used, their compositions, and the application procedures performed in the study

Adhesive System	Type	Composition	Application
Clearfil S3 Bond Universal^a^ (Kurary Noritake)	Universal multi-mode adhesive (SE1)	-MDP, Bis-GMA, HEMA, hydrophilic aliphatic dimethacrylate, colloidal silica, silane coupling agent, dl-camphorquinone, ethanol, water	1.Apply and rub for 20 s 2.Air blow gently for 5 s 3.Light-cure 10 sec
Clearfil SE Bond 2 (Kurary Noritake)	Two-step self-etching adhesive (SE2)	-Primer: 10-MDP, HEMA, hydrophilic dimethacrylate, water -Bonding: MDP, Bis-GMA, HEMA, hydrophobic dimethacrylate, photoinitiators, filler	1.Apply primer and rub for 20 s 2.Dry with mild air blow 3.Apply bonding 4.Air blow gently 5.Light-cure 10 se

*10-MDP* 10-methacryloyloxydecyl dihydrogen phosphate, *Bis-GMA* bisphenol A glycidyl dimethacrylate, *HEMA* 2-hydroxyethyl methacrylate

^a﻿^Also called Clearfil Universal Bond or Clearfil Tri-S Bond Universal, depending on the marketing area

The diode laser parameters used for irradiation of the testing areas in contact mode, tip to specimen’s surface, were as follows [9]: Energy per pulse = 80 mJ, Frequency = 10 Hz, Power = 0.8 W, Irradiation time = 30 S, and Total energy = 24 J over a testing area of 48 mm^2^. In the control subgroups (SE1-NL and SE2-NL), adhesives were light-cured for 20 s immediately after application, without laser treatment. This protocol followed manufacturer recommendations and established adhesive application guidelines to optimize bonding efficacy.

Hybrid ceramic inlays were bonded using a flowable superfilled composite resin (Clearfil Majesty Flow; Kurary Noritake) under a static load of 5 Kg using a calibrated loading device to maintain consistent pressure during the bonding procedure. This load was maintained until initial polymerization was completed, and each surface was light-cured for 20 s using a LED curing unit (Bluephase N; Ivoclar AG) to ensure complete polymerization and optimal bond strength (Fig. 1G-H).

After bonding, Specimens were stored in distilled water at 37 °C for 24 h, then aged by immersion in 10% sodium hypochlorite for one hour [31]. Specimens were then sectioned vertically into 0.9 mm thick beams using a low-speed diamond saw under water cooling (Isomet 4000; Buehler Ltd.). A total of 96 beams were obtained, each with a cross-sectional area of approximately 0.9 mm×0.9 mm (Fig. 1I-K).

Each beam consisted of half hybrid ceramic inlay and half dentin, prepared using a non-trimming technique as previously described [32]. Each beam was bonded to the testing jig using cyanoacrylate adhesive, positioned at least 1 mm away from the adhesive interface. The jig was mounted on a universal testing machine (Model 3345; Instron Corp.) equipped with a 500 N load cell. A tensile load was applied at a crosshead speed of 0.5 mm/min until failure occurred (Fig. 1L). Bond strength was calculated in megapascals (MPa) by dividing the load at failure by the cross-sectional bonding area. Multiple beams were obtained from each tooth to enhance data reliability. Microtensile bond strength (µTBS) testing followed established protocols [32, 33].

**Fig. 3:**
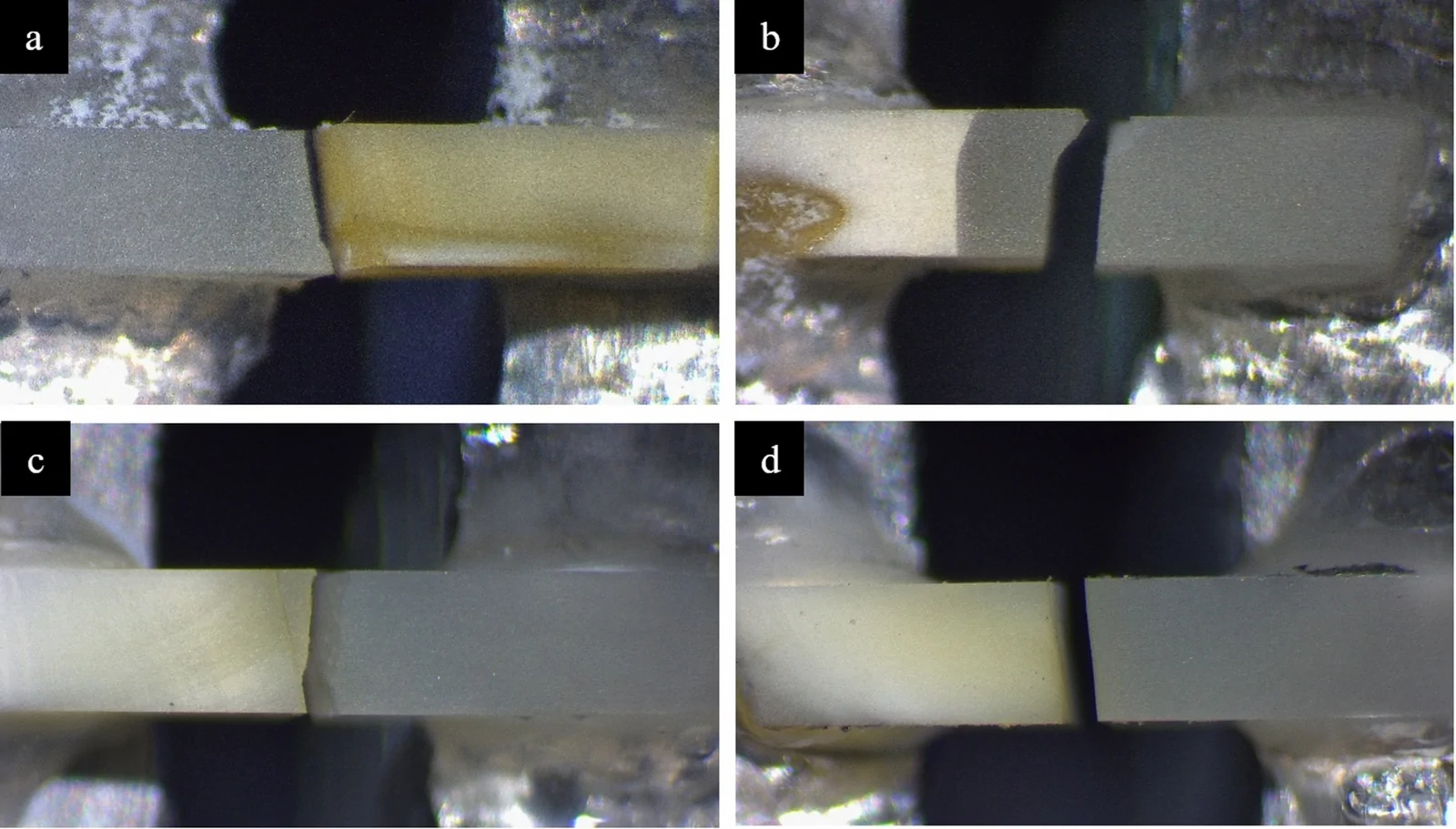
Stereomicroscope images showing mode of failure after µTBS test at 30X magnification; (**a**) Adhesive failure (**b**) Cohesive failure in ceramic (**c**) Cohesive failure in dentin (**d**) Mixed failure

Each fractured beam was examined under a stereomicroscope (MA100; Nikon) at magnification 30X to assess the mode of failure. Failures were classified as follows: adhesive (A), occurring at the adhesive interface; cohesive in dentin (CD), occurring within the dentin substrate; cohesive in ceramic (CC); occurring within the hybrid ceramic block; and mixed (M), involving a combination of adhesive and cohesive failure. The percentage of each failure type was recorded (Fig. 3).

Scanning Electron Microscopy (SEM) Analysis: Representative fractured microtensile specimens from each experimental group were selected for qualitative fractographic evaluation using a field emission scanning electron microscope (FEG250 Quanta, FEI Company, Hillsboro, OR, USA.) Prior to examination, the fractured specimens were mounted on aluminum stubs and examined in backscattered electron mode at an accelerating voltage of 30 K = kV under x160 and x2000 magnifications. Low-magnification images were obtained to evaluate the overall fracture pattern, whereas high-magnification images were used to assess surface morphology and identify characteristic fractographic features. The SEM examination was performed to qualitatively characterize the fractured surfaces and provide representative images supporting the fracture analysis of the tested groups [28].

Numerical data were assessed for normality by examining their distribution and applying the Kolmogorov-Smirnov and Shapiro-Wilk tests. Microtensile bond strength values exhibited a non-normal (nonparametric) distribution and were expressed as median, range, mean, and standard deviation (SD). The Mann-Whitney U test was used to evaluate the effect of laser application and adhesive system type on microtensile bond strength. Failure mode data were reported as frequencies and percentages and compared using Fisher’s Exact test. The significance level was set at α = 0.05. Statistical analyses were performed using statistical software (IBM SPSS Statistics for Windows, Version 23.0; IBM Corp).

## Results

Laser irradiation resulted in a statistically significant increase in microtensile bond strength compared to non-irradiated controls in both adhesive systems (Self-etch 1: *P*=.047, effect size = 1.612; Self-etch 2: *P*=.028, effect size = 1.926) (Table 2).

**Table 2:** Descriptive statistics and Mann-Whitney U test results comparing microtensile bond strength (µTBS) with and without laser irradiation

Adhesive Type	Laser (*n* = 5)		No Laser (*n* = 5)		*P*-value	Effect size (d)
	Median (Range)	Mean (SD)	Median (Range)	Mean (SD)		
SE1	13.7 (11.7, 24.9)	16.3 (5.6)	7.7 (1.5, 17)	8.8 (5.6)	0.047^*^	1.612
SE2	22 (17.6, 24.6)	21.3 (3.1)	12 (7, 19.1)	12.5 (5.4)	0.028^*^	1.926

*d* effect size, *SD* Standard deviation, *SE1* Self-etch one-step adhesive, *SE2* Self-etch two-step adhesive

^*^Significant at *P*≤.05

No statistically significant difference in bond strength was observed between the two self-etch adhesives, regardless of laser application (With laser: *P*=.251, effect size=0.780; Without laser: *P*=.600, effect size=0.335) (Table 3).

**Table 3:** Descriptive statistics and Mann-Whiteny U test results comparing microtensile bond strength (µTBS) of the two adhesive types

Laser irradiation	SE1 (*n* = 5)		SE2 (*n* = 5)		*P*-value	Effect size (d)
	Median (Range)	Mean (SD)	Median (Range)	Mean (SD)		
Laser	13.7 (11.7, 24.9)	16.3 (5.6)	22 (17.6, 24.6)	21.3 (3.1)	0.251	0.78
No Laser	7.7 (1.5, 17)	8.8 (5.6)	12 (7, 19.1)	12.5 (5.4)	0.600	0.335

*SD* Standard deviation, *d* effect size, *SE1* Self-etch one-step adhesive, *SE2* Self-etch two-step adhesive

There was no statistically significant difference between failure modes in different groups (*P*=.167, Effect size=0.477) (Table 4).

**Table 4:** Descriptive statistics and results of Fisher’s exact test for comparison between failure modes

Failure mode	SE1-L		SE2-L		SE1-NL		SE2-NL		*P*-value	Effect size (v)
	*n*	%	*n*	%	*n*	%	*n*	%		
Adhesive	2	40	1	20	4	80	0	0	0.167	0.477
Cohesive	1	20	2	40	0	0	1	20		
Mixed	2	40	2	40	1	20	4	80		

*SE1-L* Self-etch one-step adhesive laser-irradiated, *SE2-L* Self-etch two-step adhesive laser-irradiated, *SE1-NL* Self-etch one-step adhesive non-laser-irradiated, *SE2-NL* Self-etch two-step adhesive non-laser-irradiated, v effect size

### Scanning electron microscopy (SEM) fractographic analysis

**Fig. 4:**
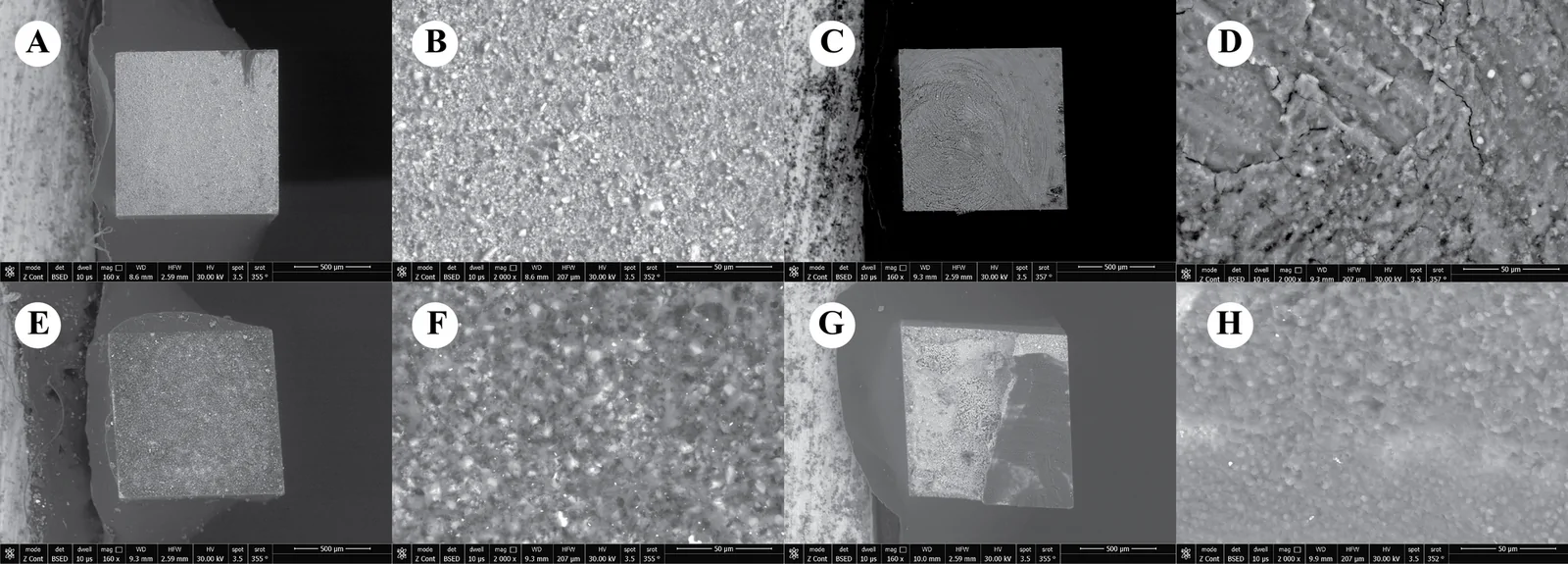
Representative scanning electron microscopy (SEM) micrographs of fractured microtensile specimens following bond strength testing. Low-magnification (160x) and high-magnification (2000x) micrographs were obtained for each experimental group. **A**,** B** SE1-NL (one-step self-etch adhesive without diode laser); **C**,** D** SE1-L (one-step self-etch adhesive with diode laser); **E**,** F** SE2-NL (two-step self-etch adhesive without diode laser); **G**,** H** SE2-L (two-step self-etch adhesive with diode laser). Low-magnification images illustrate the overall fracture morphology, whereas high-magnification images demonstrate representative surface topography of the fractured specimens. SEM examination was performed on fractured surfaces following mechanical testing; therefore, the images depict post-fracture surface morphology rather than intact adhesive interfaces

Representative SEM micrographs of the fractured microtensile specimens are presented in Fig. 4. Low-magnification (x160) images provided an overview of the fracture pattern, whereas high-magnification (x2000) images allowed qualitative assessment of the fractured surface morphology.

The SE1-NL specimens (Fig. 4A, B) exhibited a relatively homogeneous and compact fracture surface with a generally smooth and uniform topography. At higher magnification, the fractured surface demonstrated fine exposed filler particles embedded within a relatively uniform resin matrix without obvious porous regions or discernible adhesive remnants, consistent with a predominantly adhesive fracture pattern.

The SE1-L specimens (Fig. 4C, D) demonstrated a comparatively rougher and more heterogeneous fracture morphology than the corresponding non-laser group. High-magnification images revealed an irregular surface characterized by pronounced microrelief, exposed mineral and filler particles, and occasional microcracks generated during fracture. These qualitative features indicate a more complex fracture pattern and are consistent with the higher microtensile bond strength observed in laser-treated specimens.

The SE2-NL specimens (Fig. 4E, F) exhibited a relatively uniform fracture surface at low magnification, at x2000 magnification, the fractured surface appeared compact with evenly distributed exposed filler particles and minimal surface irregularities. No distinct porous regions, resin tags, or continuous adhesive layer could be identified, suggesting a fracture pattern predominantly occurring at the bonded interface.

The SE2-L specimens (Fig. 4G, H) displayed the most heterogeneous fracture morphology among the experimental groups. Low-magnification micrographs demonstrated distinct regions with different surface characteristics, including a compact area adjacent to a markedly rough and porous fractured region. At higher magnification, the fractured surface exhibited pronounced surface irregularities, numerous exposed filler particles, and a heterogeneous porous morphology. These qualitative observations suggest a more complex fracture involving the adhesive/resin complex rather than simple interfacial separation and are consistent with the significant higher bond strength values recorded for this group.

Overall, laser-treated specimens exhibited qualitatively rougher and more heterogeneous fracture surfaces than their corresponding non-laser controls, particularly in the SE2-L group, supporting the mechanical findings obtained from the microtensile bond strength test.

## Discussion

The durability and strength of the bond between restorative materials and dentin are crucial for the long-term success of indirect restorations like hybrid ceramic inlays [34, 35]. Self-etch adhesive systems have gained popularity due to simplified protocols and reduced technique sensitivity compared to total-etch systems. However, the bonding performance of self-etch adhesives is influenced by their formulation, particularly the number of application steps, solvent composition, hydrophilicity, and functional monomer content [9]. In the present study, Clearfil S3 Bond Universal was applied in self-etch mode as a simplified one-step adhesive strategy, whereas Clearfil SE Bond 2 represented a conventional two-step self-etch system. Simplified one-step adhesive strategies generally contain higher concentrations of hydrophilic monomers and solvents to combine conditioning, priming, and bonding into a single application, making adequate solvent evaporation and water removal critical for optimal polymerization and long-term bond durability [9, 36]. In contrast, two-step self-etch adhesives like Clearfil SE Bond 2 separate priming and bonding steps, facilitating improved solvent evaporation and formation of a more hydrophobic adhesive layer, which has been associated with greater resistance to hydrolytic degradation and more stable long-term bonding [17, 18]. Nevertheless, contemporary universal adhesives have demonstrated bond strength and clinical performance comparable to multi-step adhesives when applied according to the manufacturer’s instructions and appropriate adhesive strategies. Therefore, differences in bonding effectiveness appear to be more closely related to adhesive formulation, solvent composition, and application strategy than to the number of clinical steps alone [37, 38]. Because the diode laser in the present study was applied after adhesive placement and before light polymerization, any improvement in bond strength would be expected to arise primarily from photothermal effects on the uncured adhesive layer, including enhanced solvent volatilization, reduced monomer viscosity, and improved monomer conversion, rather than direct modification of the dentin surface. The magnitude of these effects may vary according to the physicochemical characteristics of the adhesive system, particularly its solvent composition and hydrophilic/hydrophobic balance [20, 21].

The present study demonstrated that diode laser irradiation applied after adhesive placement and before light polymerization significantly increased the microtensile bond strength of both adhesive strategies, regardless of whether a simplified one-step or conventional two-step self-etch protocol was used. These findings indicate that diode laser irradiation may enhance the adhesive performance of self-etch systems under the experimental conditions investigated. Qualitative SEM evaluation further revealed that laser-treated specimens exhibited a more heterogeneous and irregular fracture morphology than their corresponding non-laser controls, particularly in the SE2-L group, consistent with the higher bond strength values observed. However, because the SEM analysis was performed on fractured specimens after microtensile testing, these observations should be regarded as qualitative support for the mechanical findings rather than direct evidence of improved resin infiltration or hybrid layer formation.

The improvement in bond strength observed after diode laser irradiation is consistent with previous studies reporting enhanced adhesion when diode lasers are applied immediately after adhesive placement and before polymerization. El Hakim et al. demonstrated significantly improved dentin bond strength after irradiation of a two-step self-etch adhesive with a 940 nm diode laser [39]. Likewise, Al-Ashou et al. reported that diode laser application before photopolymerization significantly increased the bond strength of self-etch adhesive systems [40]. Similar improvements have also been reported for etch-and-rinse adhesive systems. Maenosono et al. and Kasraei et al., demonstrated increased dentin microtensile bond strength following irradiation of etch-and-rinse adhesives with 940 nm diode lasers [9, 41]. Naik et al. reported similar improvements for etch and rinse adhesives but at a different wavelength of diode laser 970 nm [42]. In contrast, Ayar and Kul found that active application of self-etch adhesives using a 976 nm diode laser produced bond strengths comparable to those obtained with the conventional application technique [43]. Similarly, Srikumar et al. reported that diode laser irradiation before photopolymerization did not produce a statistically significant improvement in shear bond strength with the adhesive systems evaluated, although the response varied among the different adhesive formulations [44]. These discrepancies likely reflect differences in laser wavelength, power density, irradiation protocol, adhesive formulation (particularly solvent type and concentration), bonding substrate, and testing methodology, emphasizing that the effectiveness of diode laser-assisted bonding depends on both the irradiation parameters and the physicochemical characteristics of the adhesive system [45, 46].

Unlike erbium lasers, whose interaction with dental hard tissues is dominated by ablation through strong absorption of water and hydroxyapatite, diode lasers operating in the 810–980 nm wavelength range exhibit limited absorption by dentin and are primarily absorbed by pigments and organic components [47]. Therefore, when diode laser irradiation is applied over an uncured adhesive layer, its principal effect is unlikely to involve smear layer removal, dentinal tubule opening, or modification of dentin surface roughness. Instead, the observed improvement in bond strength is more plausibly explained by photothermal effects within the adhesive layer itself. Localized heating may decrease adhesive viscosity, accelerate evaporation of residual solvents and water, improve monomer mobility, and increase the degree of monomer conversion before light activation, thereby promoting formation of a more homogeneous polymer network. This proposed mechanism is supported by Brianezzi et al., who demonstrated that irradiation of uncured simplified adhesive systems with a 970 nm diode laser significantly increased the degree of conversion without adversely affecting water sorption or water solubility, suggesting improved polymerization efficiency following laser irradiation [48]. These effects may be particularly beneficial for simplified one-step adhesive strategies, whose relatively high solvent and hydrophilic monomer content make efficient solvent volatilization critical for optimal polymerization and long-term bond durability [49]. Nevertheless, because neither solvent evaporation nor degree of conversion was directly measured in the present investigation, these mechanisms should be regarded as biologically plausible explanations supported by previous studies rather than definitive conclusions.

Although diode laser irradiation significantly increased µTBS in both adhesive strategies, the mean bond strength values remained below the 20–25 MPa range that has historically been suggested as desirable for reliable resin-dentin adhesion. However, this threshold should be interpreted with caution, as it was originally proposed for direct resin composite restorations rather than indirect CAD/CAM hybrid ceramic restorations tested using accelerated aging protocols [56]. In addition to the intrinsic variability associated with microtensile testing, specimen preparation may contribute to reduced bond strength values. Sectioning procedures can introduce microcracks or residual stresses within the adhesive complex, potentially resulting in premature failure and lower absolute bond strength values [28]. Likewise, although the selected laser parameters were based on previous investigations [9], different irradiation protocols may influence the balance between solvent evaporation and excessive heat generation, emphasizing the importance of parameter optimization [48]. Nevertheless, the SEM evaluation performed on representative fractured specimens did not reveal obvious morphological evidence of extensive thermal degradation or catastrophic specimen damage. In the present study, the relatively low µTBS values may also be related to the use of sodium hypochlorite aging, which is known to accelerate degradation of exposed collagen and challenge the durability of the adhesive interface [50, 51]. Therefore, although the absolute bond strength values were modest, the significant improvement observed after diode laser irradiation remains clinically relevant because all experimental groups were subjected to identical aging and testing conditions.

Although the laser-treated two-step adhesive strategy produced numerically higher µTBS values than the laser-treated simplified adhesive strategy, the difference was not statistically significant. This finding suggests that diode laser irradiations improved the bonding performance of both adhesive strategies under the conditions of the present study. The absence of a significant difference my reflect the similar functional chemistry shared by both adhesive systems, particularly the presence of the functional monomer 10-MDP (Table 1), which is capable of forming stable chemical bonds with residual hydroxyapatite [52–54]. Nevertheless, the two-step adhesive demonstrated a consistent numerical advantage that may be attributed to its separate priming and bonding procedures, allowing more efficient solvent evaporation and formation of a relatively hydrophobic adhesive layer [55–57]. Conversely, the simplified adhesive strategy combines conditioning, priming, and bonding into a single application, resulting in a more hydrophilic adhesive layer that is generally more susceptible to residual solvent retention and hydrolytic degradation. The present findings therefore suggest that diode laser irradiation may partially compensate for these physicochemical limitations by enhancing solvent volatilization and adhesive polymerization, although the numerical superiority of the two-step adhesive should be interpreted cautiously because of the limited sample size [58–60].

Failure mode analysis revealed no statistically significant differences among groups with adhesive, cohesive and mixed failures distributed across all groups. The predominance of adhesive and mixed failures is consistent with the relatively low bond strength values recorded and suggests that failure generally occurred at or near the adhesive interface. Similar distributions have been reported in previous microtensile bond strength studies evaluating self-etch adhesive systems [28, 45, 52]. The absence of significant differences among the groups indicates that neither the adhesive strategy nor diode laser irradiation substantially altered the predominant fracture pattern under the present experimental conditions.

Representative SEM examination provided qualitative characterization of the fractured specimens following microtensile testing. Laser-treated specimens generally exhibited more irregular and heterogeneous fracture topography than their corresponding non-laser controls, particularly in the SE2-L group, where distinct fracture regions and rougher fracture morphology were observed. These qualitative observations are consistent with the higher µTBS values obtained after laser irradiation and suggest a more complex fracture pattern involving the adhesive/resin complex. However, because SEM evaluation was performed on fractured specimens after testing, the images primarily represent post-fracture surface morphology rather than the intact adhesive interface. Consequently, they cannot directly demonstrate resin tag formation, hybrid layer quality, or adhesive penetration into dentinal tubules. More comprehensive characterization of the adhesive interface would require complementary techniques such as a cross-sectional SEM, transmission electron microscopy, confocal laser scanning microscopy, nanoleakage analysis, or in situ zymography [45, 52].

Several limitations of the present investigation should be acknowledged. First, the sample size was limited, although it was determined by an a priori power analysis and is consistent with previous in vitro µTBS investigations [39, 40]. Second, only short-term accelerated aging by sodium hypochlorite immersion was performed, without thermocycling, prolonged water storage, or cyclic mechanical loading. Third, the proposed mechanism of laser action was inferred from the literature, as neither solvent evaporation, degree of monomer conversion, nor pulpal temperature changes were directly evaluated. Finally, the SEM analysis was qualitative and performed only on representative fractured specimens rather than intact adhesive interfaces. Future studies should combine mechanical testing with physicochemical characterization of the adhesive layer, evaluate thermal effects of diode laser irradiation on pulpal tissues, and investigate the long-term durability of laser-assisted adhesive protocols under clinically relevant aging conditions.

Additionally, because diode laser irradiation required approximately 30 s before light polymerization, the control groups did not include an equivalent delayed-cure interval. Although this sequence reflected the intended clinical laser protocol, the independent effect of the additional waiting time on bond strength could not be isolated. Future studies incorporating delayed-curing control groups are warranted to distinguish the influence of laser irradiation from that of polymerization timing.

Overall, the present findings partially rejected the proposed null hypotheses. Diode laser irradiation applied before adhesive polymerization significantly improved the microtensile bond strength of both simplified one-step and conventional two-step self-etch adhesive strategies to dentin, leading to rejection of the first null hypothesis. However, no statistically significant difference was observed between the adhesive strategies; therefore, the second null hypothesis was not rejected. Although the two-step adhesive exhibited numerically higher bond strength values, these differences should be interpreted cautiously because of the limited sample size and the in vitro nature of the study. These findings suggest that diode laser irradiation represents a promising adjunct to adhesive procedures; however, further investigations are required before definitive clinical recommendations can be established.

## Conclusion

Based on the findings of this in vitro study, the following conclusions were drawn:

1. Diode laser irradiation applied after adhesive placement and before light polymerization significantly increased the microtensile bond strength of both simplified one-step and conventional two-step self-etch adhesive strategies when bonding hybrid ceramic restorations to dentin.
2. Although the laser-treated two-step adhesive demonstrated numerically higher bond strength values, no statistically significant difference was observed between the two adhesive strategies following laser irradiation.
3. Within the limitations of this in vitro study, diode laser irradiation appears to be a promising adjunct to self-etch adhesive protocols. However, further investigations evaluating adhesive polymerization, interfacial morphology, pulpal safety, long-term durability, and clinical performance are required before routine clinical implementation can be recommended.

## Data Availability

The data sets used and/or analyzed during the current study are available from the corresponding author upon reasonable request.

## Abbreviations

µTBS: Micro-tensile bond strength
SE1: One-step self-etch adhesive
SE2: Two-step self-etch adhesive
SE1-L: One-step self-etch adhesive laser-irradiated subgroup
SE2-L: Two-step self-etch adhesive laser-irradiated subgroup
SE1-NL: One-step self-etch adhesive non-irradiated control subgroup
SE2-NL: Two-step self-etch adhesive non-irradiated control subgroup
CAD-CAM: Computer-aided design and computer-aided manufacturing
MPa: MegaPascal
A: Adhesive mode of failure
CD: Cohesive in dentin mode of failure
CC: Cohesive in ceramic mode of failure
M: Mixed mode of failure involving a combination of adhesive and cohesive failure
SD: Standard deviation
